# Using deep learning to assist readers during the arbitration process: a lesion-based retrospective evaluation of breast cancer screening performance

**DOI:** 10.1007/s00330-021-08217-w

**Published:** 2021-08-12

**Authors:** Laura Kerschke, Stefanie Weigel, Alejandro Rodriguez-Ruiz, Nico Karssemeijer, Walter Heindel

**Affiliations:** 1 Institute of Biostatistics and Clinical Research, IBKF, University of Muenster, Schmeddingstrasse 56, 48149 Muenster, Germany; 2 Clinic for Radiology and Reference Center for Mammography Muenster, University of Muenster and University Hospital Muenster, Albert-Schweitzer-Campus 1, 48149 Muenster, Germany; 3 ScreenPoint Medical BV, Toernooiveld 300, 6525 EC Nijmegen, The Netherlands; 4 Department of Radiology and Nuclear Medicine, Radboud University Medical Center, Geert Grooteplein Zuid 10, 6525 Nijmegen, GA The Netherlands

**Keywords:** Breast cancer, Screening, Mammography, Artificial intelligence

## Abstract

**Objectives:**

To evaluate if artificial intelligence (AI) can discriminate recalled benign from recalled malignant mammographic screening abnormalities to improve screening performance.

**Methods:**

A total of 2257 full-field digital mammography screening examinations, obtained 2011–2013, of women aged 50–69 years which were recalled for further assessment of 295 malignant out of 305 truly malignant lesions and 2289 benign lesions after independent double-reading with arbitration, were included in this retrospective study. A deep learning AI system was used to obtain a score (0–95) for each recalled lesion, representing the likelihood of breast cancer. The sensitivity on the lesion level and the proportion of women without false-positive ratings (non-FPR) resulting under AI were estimated as a function of the classification cutoff and compared to that of human readers.

**Results:**

Using a cutoff of 1, AI decreased the proportion of women with false-positives from 89.9 to 62.0%, non-FPR 11.1% vs. 38.0% (difference 26.9%, 95% confidence interval 25.1–28.8%; *p* < .001), preventing 30.1% of reader-induced false-positive recalls, while reducing sensitivity from 96.7 to 91.1% (5.6%, 3.1–8.0%) as compared to human reading. The positive predictive value of recall (PPV-1) increased from 12.8 to 16.5% (3.7%, 3.5–4.0%). In women with mass-related lesions (*n* = 900), the non-FPR was 14.2% for humans vs. 36.7% for AI (22.4%, 19.8–25.3%) at a sensitivity of 98.5% vs. 97.1% (1.5%, 0–3.5%).

**Conclusion:**

The application of AI during consensus conference might especially help readers to reduce false-positive recalls of masses at the expense of a small sensitivity reduction. Prospective studies are needed to further evaluate the screening benefit of AI in practice.

**Key Points:**

*• Integrating the use of artificial intelligence in the arbitration process reduces benign recalls and increases the positive predictive value of recall at the expense of some sensitivity loss.*

*• Application of the artificial intelligence system to aid the decision to recall a woman seems particularly beneficial for masses, where the system reaches comparable sensitivity to that of the readers, but with considerably reduced false-positives.*

*• About one-fourth of all recalled malignant lesions are not automatically marked by the system such that their evaluation (AI score) must be retrieved manually by the reader. A thorough reading of screening mammograms by readers to identify suspicious lesions therefore remains mandatory.*

## Introduction

As more deep learning–based artificial intelligence (AI) mammography screening tools enter the clinical market, greater focus will be placed on scientific validation in diverse settings [1]. In digital mammography reader studies, AI demonstrated the ability to significantly reduce radiologists’ workload, improve specificity, and reach a cancer detection rate comparable to radiologists [1–6].

However, clinical validation is lacking, and it is not clear how the power of deep learning should be used to optimize practice [7]. Most of the previous works were based on retrospectively selected, cancer-enriched data sets that do not reflect the setting of a population-based screening program in practice, where cancer prevalence is much lower [8]. Translation of retrospective findings resulting from a particular experimental setting to different steps of the screening process remains mainly unclear [9]. Furthermore, only few studies took the localization accuracy of the AI system into account, to verify that a location-level false-positive and false-negative finding on the same mammogram do not result in a true-positive rating on the image level [6, 8, 10].

False-positive results are one negative side effect of mammography screening [11, 12]. As most screening examinations are finally negative, AI might be useful to increase the specificity and positive predictive value of recall (PPV-1) by reducing reader-induced false-positive results [10]. In Germany, independent double-reading with arbitration results in up to 25% of all examinations being discussed in a consensus conference by the first and second reader together with a third reader. Most of the cases discussed are dismissed during the arbitration process leading to recommendation of regular biennial screening. Finally recall recommendation is given in 41 out of 1000 women screened, to diagnose 6 women with breast cancer [13, 14]. In contrast, in the Netherlands, a lower recall rate of 21 out of 1000 women screened is reported together with a comparable cancer detection rate [15].

There is no evidence from the literature how AI may help guiding human decision during the arbitration process on whether a suspicious mammographic lesion requires further diagnostic workup. Before using AI prospectively with the aim to lower false-positive recalls, it seems mandatory to scientifically evaluate its performance retrospectively in a real-world dataset of consecutive recall examinations from a population-based screening program. The subset of women that undergo recall assessments provides particularly high-quality data with accurate lesion-based ground-truth labels due to further imaging procedures, histologic workup (if indicated) and 2-year follow-up for interval cancers.

The purpose of the study is to evaluate if a deep learning–based AI system can discriminate benign from malignant mammographic screening abnormalities that led to recall recommendation following double-reading with arbitration, to reduce assessments of benign findings and increase the PPV-1.

## Materials and methods

This retrospective study was conducted with consecutive digital mammographic screening examinations obtained between 2011 and 2013 at the Reference Screening Unit Muenster, Germany. The study was approved by the local ethics committee (Ärztekammer Westfalen-Lippe and University of Muenster, Germany). Informed consent was not required for the evaluation of internal anonymized data.

### Screening setting

The national mammography screening program in Germany is based on European guidelines [13]. The target population includes women aged 50 to 69 years who are invited biennially. The program comprises masked independent readings of two-view 2D digital mammograms by two certified physician readers. Qualifications have been described in detail elsewhere [16]. Prior to recall recommendation, suspicious findings of one or both readers have to be discussed in a consensus conference (arbitration process) by both readers together with a third physician, who performs the centrally organized assessment [17].

### Study setting

During the screening period 2011 to 2013, a total of 41,722 digital mammography examinations were obtained by two vendors (MicroDose Mammography (L30), Philips Medical Systems; Inspiration, Siemens Healthineers). Five readers with more than five years of experience in breast imaging performed the independent double readings. Recall recommendation was finally given at the consensus conference in 2957 women. Screening data of recalled women (including breast density, radiological lesion features assessed at the consensus conference and assessment reports), documented at the time of screening, were retrospectively derived from the screening documentation software MaSc.

**Fig. 1:**
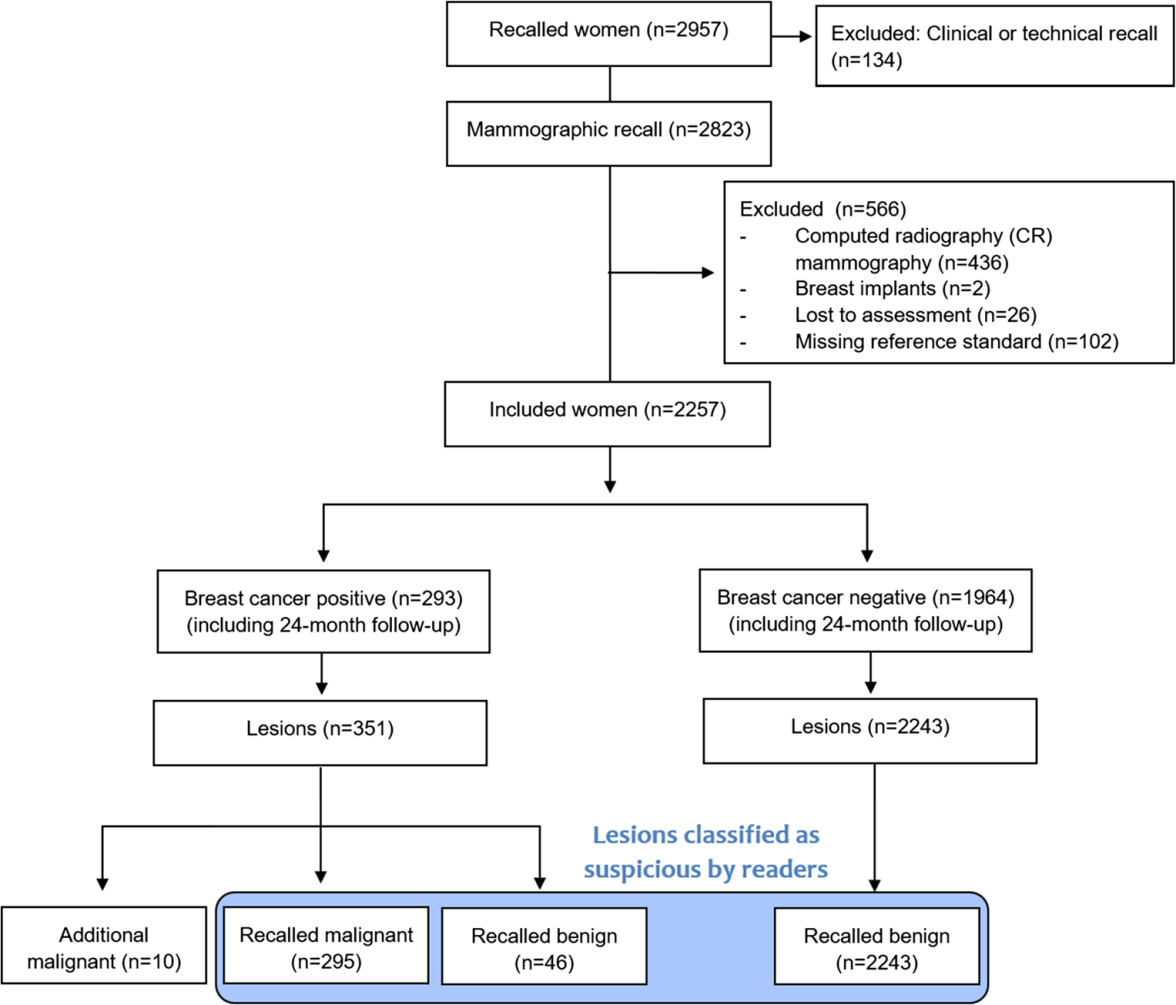
Flow chart of screening examinations selected for the study. The ground truth in terms of cancer presence was determined based on histopathology and/or 24-month follow-up. Recalled malignant: malignant lesion detected by independent double reading and arbitration with recall recommendation (i.e., reader true-positive); recalled benign: benign lesion suspicious for malignancy after independent double reading with arbitration (i.e., reader false-positive); additional malignant: malignant lesion detected during assessment or 24-month interval after negative assessment, not marked for recall during consensus conference

One hundred thirty-four (0.3%) of all recall recommendations were based on clinical or technical reasons and were excluded from this study. Two thousand eight hundred twenty-three (6.7%) recalls resulted from mammographic abnormalities. Of the 2,823 mammographic recalls, 566 were excluded due to computed radiography (CR) technique (*n* = 436), breast implants (*n* = 2), loss to assessment (*n* = 26), or missing reference standard information (RSI) (*n* = 102), resulting in 2257 examinations included in the study (Fig. 1). The RSI included assessment participation with bilateral ultrasound plus, if indicated, additional mammographic views, the completion of follow-up examinations and invasive procedures (e.g., additional breast surgery after minimally invasive tissue sampling), and the information of the cancer registry on interval cancers. Interval cancers were defined as invasive breast cancers and ductal carcinoma in situ (DCIS) occurring within 24 months after negative screening participation. Histological evaluation after minimal invasive biopsies within the screening program were performed by three certified pathologists [16], with 5 to > 15 years of experience in breast pathology.

### Performance evaluation of human readers and AI

For study purposes, recalled mammographic lesions were defined malignant (i.e., reader true-positive), if a histologically confirmed invasive breast cancer or DCIS was diagnosed at the specific lesion location (defined at the consensus conference) within the screening program (recall assessment) or the 24-month interval after a negative assessment. Otherwise, recalled lesions were defined benign (i.e., reader false-positive).

**Fig. 2:**
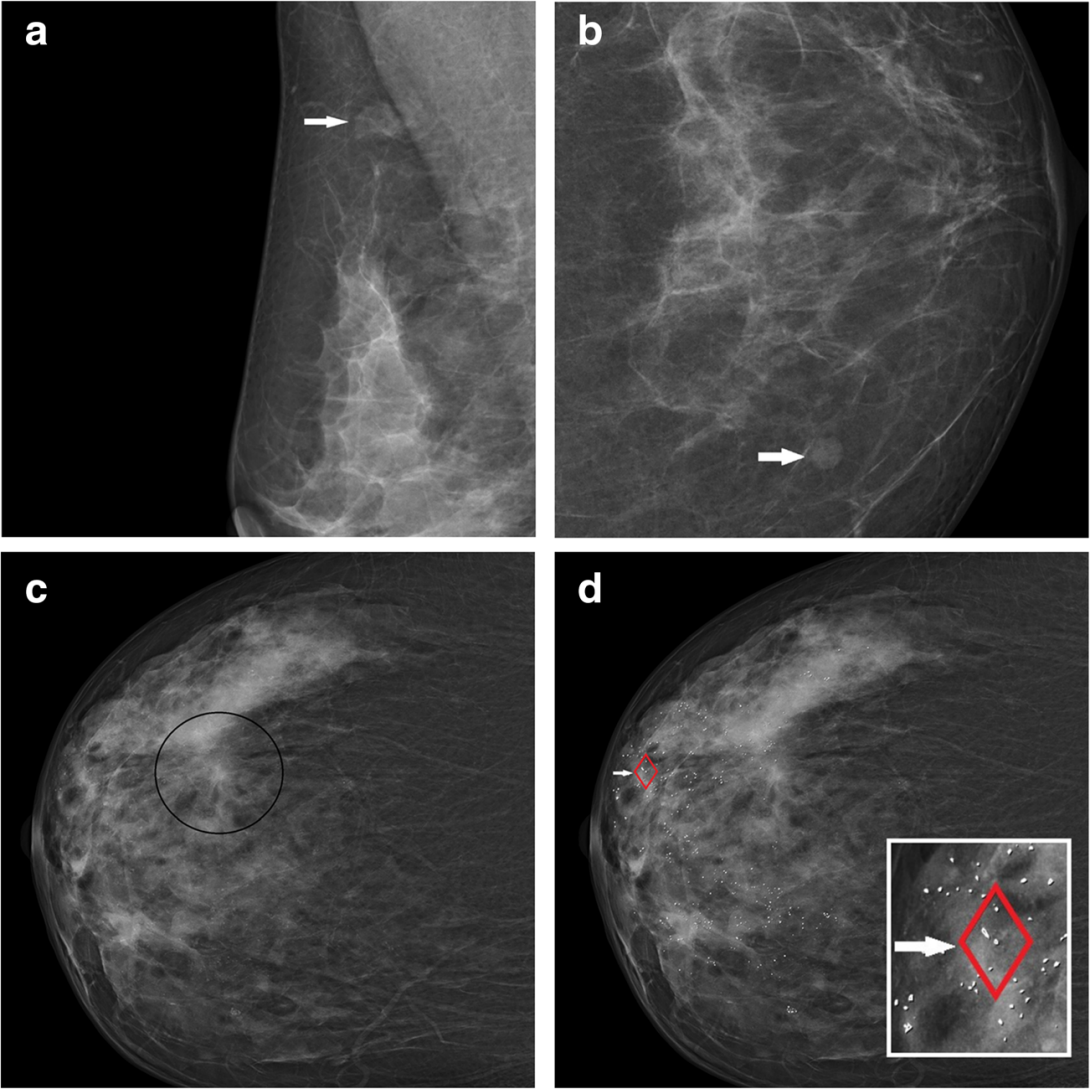
Full-field digital screening mammographic views of two breast cancer–negative women (**a**, **b**) and a breast cancer–positive woman (**c**–**d**) from the study sample. **a** Recalled density depicted by the right medio-lateral-oblique view of the screening mammogram. Assessment confirmed a benign focal asymmetry (reader false-positive). The software did not mark the lesion and did not display a lesion-specific score. The score was therefore evaluated as 0. **b** Recalled round mass, indistinct margin, located in the medial quadrants of the left breast shown in the cranio-caudal view. Assessment including minimal invasive biopsy confirmed a fibroadenoma (reader false-positive). The software did not mark the lesion and did not display a lesion-specific score (evaluated as 0). **c** Recalled architectural distortion located in the lateral quadrants of the right breast shown in the cranio-caudal view. Assessment confirmed an invasive breast cancer (no special type, pT1c, pN1a, cM0, G1) (reader true-positive). The software missed the invasive cancer (lesion-specific score evaluated as 0), but instead marked amorphous calcifications (not recalled by readers and therefore not included in the evaluation) related to benign changes (**d**). The lesion-specific score of the calcification was 42 resulting in a high overall score of 9

Therefore, each recalled lesion was relocated by one of the screening readers in the corresponding screening mammograms based on the original screening documentation. The AI software Transpara (*version 1.5.0, ScreenPoint Medical*) was used to obtain a region-based integer-valued score (0, 27, 28, …, 95) for each recalled lesion. This was realized either by automatically placed lesion marks or, if not automatically marked by AI, by manually clicking on the lesion in order to obtain a circle around the lesion (varying lesion-dependent in size). The software is a commercial product using deep learning convolutional neural networks to automatically detect regions suspicious of breast cancer in mammograms [3, 4, 18]. Version 1.5.0 of the software was trained and tested based on a proprietary database of approximately 200,000 2D mammograms (including 10,000 malignant and 5,000 benign), including images from devices of five different vendors (Hologic, Siemens Healthineers, GE Healthcare, Philips, Fujifilm Healthcare) collected at multiple institutions across Europe, the USA, and Asia*.* For each automatically detected finding in a mammogram, the software (internally) assigns a score from 1 to 100. For AI findings that are graded 95–100, a score of 95 is displayed for the user. For AI findings that are graded 1–26 and all regions without AI findings, no score is displayed. Recalled lesions for which no score was displayed were therefore assigned a value of 0 (Fig. 2a–c). If a lesion was visible in both views (cranio-caudal, medio-lateral-oblique), the higher score was used for the evaluation. Based on the (internal) region scores, the software always displays an overall score between 1 and 10 for the whole examination (not considered in the evaluation). We performed a lesion-based analysis using the specific region score of each recalled lesion to account for potential location-level false-positives and false-negatives (Fig. 2c–d). For each possible cutoff 0, 1, 27,..., 96, recalled malignant and recalled benign lesions for which the AI score exceeded the respective cutoff were evaluated as AI true-positives and false-positives, respectively. Recalled malignant and recalled benign lesions for which the score did not exceed the cutoff were evaluated as AI false-negatives and true-negatives, respectively.

Further, malignant lesions of recalled patients that were additionally detected during assessment or the 24-month interval after a negative assessment (independent of a visibility on the screening mammogram) were generally evaluated as false-negatives in all sensitivity calculations (both under human and AI reading).

As each recalled women can be affected by more than one mammographic lesion, false-positives can occur both in breast cancer–negative and in breast cancer–positive women.

### Statistical analyses

Following the free-response paradigm [19], the primary endpoints of the study were:

- the proportion of women without any false-positive ratings out of all women (including women with true-positive lesions), termed non-false-positive rate (non-FPR), and
- the related lesion-specific sensitivity, defined as proportion of detected malignant lesions (i.e., true-positives) out of all malignant lesions.

Notice that the non-FPR is in principle a specificity measurement adapted to the situation of (possibly) multiple lesions per patient. The primary endpoints were estimated as a function of the classification cutoff for the AI score. A two-sided 95% confidence interval (CI) according to Tango [20] was obtained to estimate the difference in the non-FPRs resulting under human and targeted AI reading based on the cutoff of 1 that led to the lowest decrease in the lesion-specific sensitivity, while increasing the non-FPR under AI reading as compared to human reading. The non-FPRs were compared using McNemar’s Test. Furthermore, a 95% CI for the difference in the lesion-specific sensitivities was calculated by nonparametric bootstrap. A 95% CI for the difference in the PPV-1 was determined using the Kosinski method [21]. Sensitivity and non-FPR were additionally analyzed in the two subgroups of women with reader-identified mass- or calcification-related lesions (with these lesions potentially showing additional lesion type characteristics, e.g., calcification + asymmetry). Women with at least one lesion not presenting as mass or calcification, respectively, were not included in the subgroups.

Statistical analyses were performed with R, version 4.0.2. *p* values were interpreted in Fisher’s sense, representing a metric weight of evidence against the null hypothesis of no effect. *p* < .05 was considered noticeable.

## Results

In total, 2257 women recalled for further assessment of 295 malignant and 2289 benign mammographic lesions were included (Fig. 1). Nine of these women were affected by 10 additional malignant lesions, 5 of which were detected during assessment, and 5 of which were detected as interval cancers during the 24-month follow-up interval (outside the screening program). Out of all recalled women, 293 were breast cancer positive (13.0%) and 1964 were breast cancer negative (87.0%). A screening-detected breast cancer (i.e., detection of at least one malignant lesion already identified during the arbitration process or during assessment) was diagnosed in 288 of the breast cancer positive women. Four of these women (2 of which were recalled for a malignant lesion, 2 of which were recalled for benign lesions only) were affected by at least one additional malignant lesion detected during assessment. An interval cancer occurred in 5 women, which were all recalled for a benign lesion only. Of the 2584 recalled lesions, 295 were malignant (11.4%), corresponding to 286 of 293 breast cancer positive women, and 2289 (88.6%) were benign, corresponding to 1,964 women without and 43 women with breast cancer. Patient- and lesion-specific characteristics are summarized in Tables 1 and 2. Four of five malignant lesions diagnosed during interval (80%) were considered to be true interval cancers without signs of malignancy on the screening mammogram (Table 3).

**Table 1:** Characteristics of the study sample and mammography data included in the study

**Variable**	**All recalled women included**	**Women with screening-detected breast cancer**	**Women without breast cancer (incl. 2-year follow-up)**	**Women with an interval cancer (in 2-year follow-up)**
**Number (*N*)**	2257	288	1964	5
**Age (years)**				
50–54	1180 (52.3)	86 (29.9)	1093 (55.7)	1 (20.0)
55–59	409 (18.1)	67 (23.3)	340 (17.3)	2 (40.0)
60–64	384 (17.0)	70 (24.3)	314 (16.0)	0 (0.0)
65–69	284 (12.6)	65 (22.6)	217 (11.0)	2 (40.0)
**Screening round**				
First	949 (42.0)	71 (24.7)	877 (44.7)	1 (20.0)
Subsequent	1308 (58.0)	217 (75.3)	1087 (55.3)	4 (80.0)
**Mammography device**				
Philips Microdose (L30)	1512 (67.0)	190 (66.0)	1318 (67.1)	4 (80.0)
Siemens Inspiration	745 (33.0)	98 (34.0)	646 (32.9)	1 (20.0)
**Breast density category analogue to BI-RADS 4th ed.**				
1	38 (1.7)	7 (2.4)	31 (1.6)	0 (0.0)
2	588 (26.1)	85 (29.5)	502 (25.6)	1 (20.0)
3	1491 (66.0)	172 (59.7)	1315 (67.0)	4 (80.0)
4	140 (6.2)	24 (8.3)	116 (5.9)	0 (0.0)
**Surgical therapy**				
Breast conserving therapy	–	248 (86.1)	–	0 (0.0)
Mastectomy	–	21 (7.3)	–	0 (0.0)
No surgery	–	4 (1.4)	–	0 (0.0)
Missing	–	15 (5.2)	–	5 (100)

Data are presented as absolute frequencies with percentages in parentheses

The ground truth in terms of cancer presence was determined based on histopathology and/or 24-month follow-up

**Table 2:** Characteristics of the mammography lesions of the study sample

**Variable**	**All recalled lesions**	**Recalled benign lesions**	**Recalled malignant lesions**	**Malignant lesions detected during assessment or interval**
**Number (*N*)**	2584	2289	295	10
**Category analogue to BI-RADS 4th ed.**				
4a	2371 (91.8)	2221 (97.0)	150 (50.8)	–
4b	169 (6.5)	67 (2.9)	102 (34.6)	–
5	44 (1.7)	1 (0.04)	43 (14.6)	–
**Histological type**				
Invasive breast cancer	–	–	203 (68.8)	8 (80.0)
Ductal carcinoma in situ	–	–	92 (31.2)	2 (20.0)
**Findings of lesion characteristic at the consensus conference**	2985	2571	414	
Mass	1098	945	153	–
Calcifications	820	651	169	–
Asymmetry	115	101	14	–
Architectural distortion	220	160	60	–
Density	732	714	18	–

Data are presented as absolute frequencies with percentages in parentheses

Three hundred seventy-seven of the 2584 recalled lesions showed more than one lesion type characteristic at the consensus conference. Five additional malignant lesions, corresponding to 4 women, were detected during assessment (2 ultrasound detected, presenting as masses and corresponding to 2 women with additional recalled malignant mass lesion; 2 ultrasound detected and 1 MRI-detected, all without mammographic sign and corresponding to 2 women with additional recalled benign lesions only) and 5 were detected during the 24-month interval after a negative assessment (4 true interval cancer; 1 cancer with minimal sign)

**Table 3:** Characteristics of malignant lesions detected during assessment or interval and of recalled malignant lesions detected or missed by the AI system using a cutoff of 1

**Variable**	**Detected by readers and AI**	**Detected by readers and missed by AI**	**Detected during assessment**	**Detected during interval**
**Number (*N*)**	278	17	5	5
**Category analogue to BI-RADS 4th ed.**				
4a	135 (48.6)	15 (88.2)	–	–
4b	100 (36.0)	2 (11.8)	–	–
5	43 (15.5)	0 (0.0)	–	–
AI score*	82 (39.0)	0 (0.0)	–	–
≥ 1	278 (100)	0 (0.0)	–	–
< 1	0 (0.0)	17 (100)	–	–
**Marked by AI lesion localization function**				
Yes	223 (80.2)	0 (0.0)	–	–
No	55 (19.8)	17 (100)	–	–
**Findings of lesion characteristic at the consensus conference**	391	23		
Mass	151	2	–	–
Calcifications	157	12	–	–
Asymmetry	11	3	–	–
Architectural distortion	56	4	–	–
Density	16	2	–	–
**Interval cancer classification**				
True	–	–	–	4 (80.0)
Minimal sign	–	–	–	1 (20.0)
**T (tumor)**				
Tis (in situ)	86 (30.9)	7 (41.2)	2 (40.0)	0 (0.0)
T1	165 (59.4)	9 (52.9)	3 (60.0)	5 (100)
T2	21 (7.6)	0 (0.0)	0 (0.0)	0 (0.0)
T3	1 (0.4)	0 (0.0)	0 (0.0)	0 (0.0)
T4	1 (0.4)	0 (0.0)	0 (0.0)	0 (0.0)
Unknown	4 (1.4)	1 (5.9)	0 (0.0)	0 (0.0)
**N (lymph nodes)**				
N0	231 (83.1)	15 (88.2)	4 (80.0)	5 (100)
N1	32 (11.5)	1 (5.9)	1 (20.0)	0 (0.0)
N2	4 (1.4)	0 (0.0)	0 (0.0)	0 (0.0)
N3	1 (0.4)	0 (0.0)	0 (0.0)	0 (0.0)
Unknown	10 (3.6)	1 (5.9)	0 (0.0)	0 (0.0)
**M (metastasis)**				
Negative	266 (95.6)	16 (94.1)	5 (100)	5 (100)
Positive	2 (0.7)	0 (0.0)	0 (0.0)	0 (0.0)
Unknown	10 (3.6)	1 (5.9)	0 (0.0)	0 (0.0)

Data are presented as absolute frequencies with percentages in parentheses

Ninety-eight lesions detected by readers and AI, and 5 lesions detected by readers and missed by AI showed more than one lesion type characteristic at the consensus conference. Of the 5 lesions detected during assessment, 2 were ultrasound detected, presenting as masses and corresponding to 2 women with additional recalled malignant mass lesion. The remaining 3 lesions were ultrasound- (2) and MRI-detected (1), all without mammographic abnormality and corresponding to 2 women with additional benign findings only. Four out of 5 malignant lesions diagnosed during the 24-month interval after a negative assessment were true interval cancers not visible on the screening mammogram. For lesions with neoadjuvant therapy (*n* = 12), the clinical TNM classifications were used

*Data are median (interquartile range)

### Performance of original human reading

The lesion-specific sensitivity of independent double reading with arbitration was 96.7% (295 of 305 malignant lesions). In total, 11.1% (250 of 2257 women) had no false-positive rating. The PPV-1 was 12.8% (288 of 2257 women) (Table 4).

**Table 4:** Performance measures of original human reading and targeted AI reading using a cutoff of 1 in the total cohort and in subgroups

**Performance measure**	**Original human reading**	**Targeted AI reading**	**Difference (95% CI)**
Total cohort (*n* = 2257 women)			
Lesion-specific sensitivity	96.7%	91.1%	5.6% (3.1%, 8.0%)
Non-FPR	11.1%	38.0%	26.9% (25.1%, 28.8%)
PPV-1	12.8%	16.5%	3.7% (3.5%, 4.0%)
Subgroup of women with mass-related lesions (*n* = 900 women)			
Lesion-specific sensitivity	97.8%	96.4%	1.4% (0.0%, 3.5%)
Non-FPR	14.2%	36.7%	22.4% (19.8%, 25.3%)
PPV-1	14.9%	18.9%	4.0% (3.7%, 4.4%)
Subgroup of women with calcification-related lesions (*n* = 660 women)			
Lesion-specific sensitivity	98.1%	91.7%	6.4% (3.1%, 10.8%)
Non-FPR	21.2%	34.8%	13.6% (11.2%, 16.5%)
PPV-1	22.9%	25.1%	2.3% (1.7%, 2.8%)

Lesion-specific sensitivity: proportion of detected malignant lesions; non-FPR: proportion of women without any false-positive ratings out of all women; PPV-1: positive predictive value of recall; 95% CI: 95% confidence interval

### Performance of targeted AI reading

**Fig. 3:**
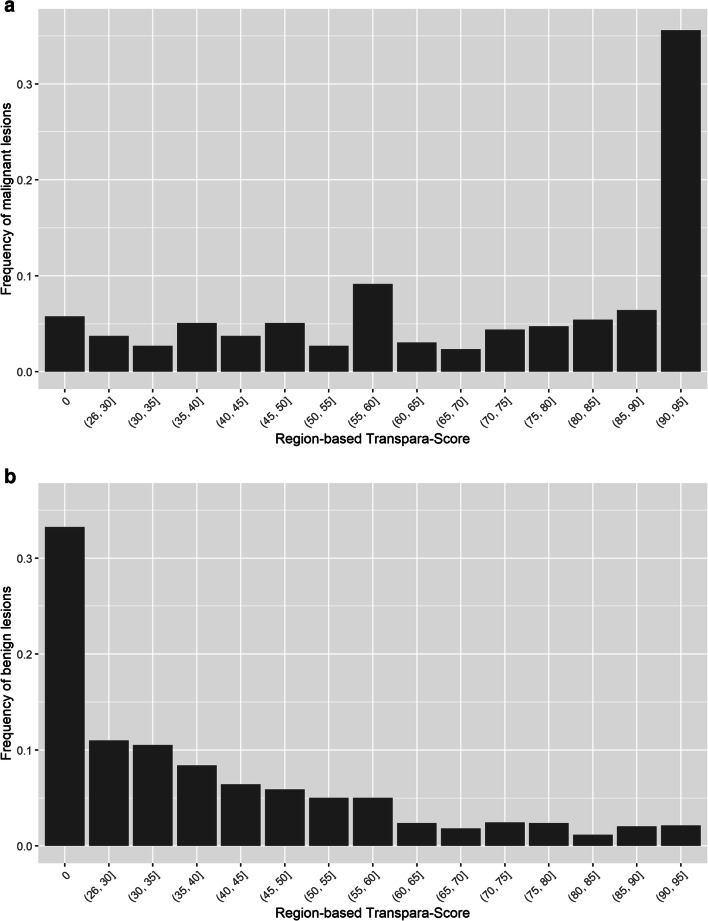
Distribution of recalled malignant (**a**) and recalled benign (**b**) lesions as a function of the AI score, representing the likelihood of breast cancer (0, 27, 28, …, 95).

The distribution of recalled malignant and benign lesions as a function of the AI score is shown in Fig. 3. Of the 295 reader-detected malignant lesions, 105 (35.6%) were rated with a score exceeding 90, whereas 278 (94.2%) were rated higher than 0. The remaining 17 reader-detected malignant lesions (5.8%) were all rated with a score of 0 (see, e.g., Fig. 2c). In contrast, 761 (33.2%) of the 2289 recalled benign lesions (reader false-positives) were assigned a score of 0 (see, e.g., Fig. 2a–b) and 1528 (66.8%) were rated higher than 0. When activating the lesion localization function of the AI system, 223 of the 295 recalled malignant lesions (75.6%) and 522 of the 2289 recalled benign lesions (22.8%) were automatically marked.

**Fig. 4:**
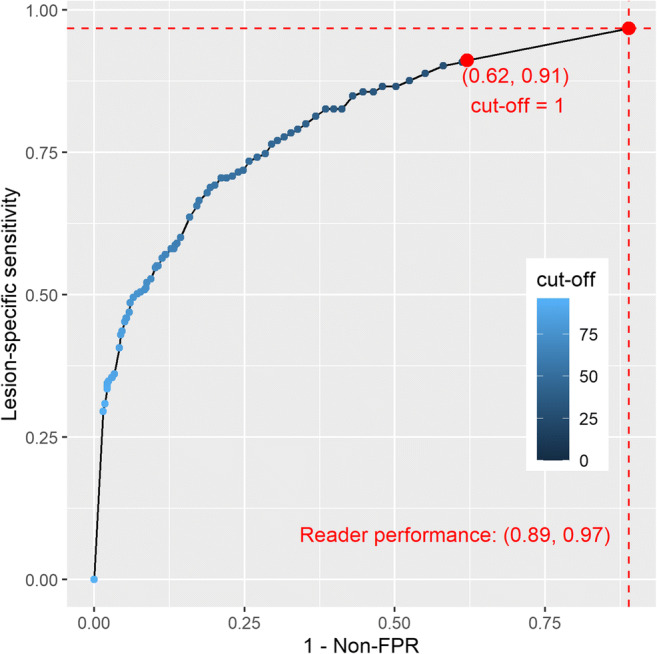
Diagnostic performance of the AI system as a function of the classification cutoff for the AI score (0, 27, 28, …, 95). For each cutoff, the *x*-axis displays the proportion of women with at least one false-positive (i.e., recalled benign lesion with a score ≥ cutoff) out of all (2,257) women, whereas the *y*-axis shows the corresponding true-positive rate (i.e., proportion of recalled malignant lesions with a score ≥ cutoff out of all (305) malignant lesions). Point coordinates corresponding to the cutoff that yield the lowest decrease in sensitivity are shown in parentheses. Since 10 malignant lesions were not detected by double-reading with arbitration, the end point (1, 1) cannot be reached.

The lesion-specific sensitivity and corresponding non-FPR of the AI system resulting under varying cutoffs for the AI score are shown in Fig. 4. Consistent with the results from Fig. 3, a cut off of 1 yielded the lowest decrease in the lesion-specific sensitivity from 96.7% (295 of 305 malignant lesions) to 91.1% (278 of 305 lesions), while increasing the non-FPR from 11.1% (250 of 2257 women) to 38.0% (857 of 2257 women) (difference: 26.9%; 95% CI 25.1–28.8%; *p* < .001) as compared to human reading (Table 4). The non-FPR improvement achieved by AI translated into an increase of the PPV-1 from 12.8% (288 out of 2257 women) to 16.5% (272 of 1649 women) (Table 4) and a reduction of 30.1% (592 of 1969 women) of reader-induced false-positive recalls.

Of the 295 reader-detected malignant lesions, 17 (5.8%) were missed by the AI system, corresponding to 17 women. One of these women was affected by a further malignant lesion detected by AI, resulting in 16 breast cancer diagnoses that were lost on the patient level. Missed lesions included 7 (41.2%) DCIS (7.6% of all recalled DCIS lesions) and 10 (58.8%) invasive cancers (5.2% of all recalled invasive lesions) and were primarily characterized by mammographic signs of lower suspicion of malignancy. The majority of these lesions (70.6%) presented as calcifications (Table 3).

AI performance was highest in the subgroup of 900 women (39.9%) with mass-related lesions (lesion-specific sensitivity of original vs. AI reading: 97.8% vs. 96.4% [135 vs. 133 of 138 lesions]; non-FPR: 14.2% vs. 36.7% [128 vs. 330 of 900 women]), while it was low in the subgroup of 660 (29.2%) women with calcification-related lesions (lesion-specific sensitivity: 98.1% vs. 91.7% [154 vs. 144 of 157 lesions]; non-FPR: 21.2% vs. 34.8% [140 vs. 230 of 660 women] original vs. AI reading) (Table 4).

## Discussion

We assessed the diagnostic performance of a deep learning artificial intelligence (AI) system to predict malignancy of 2D mammographic lesions that led to a recall recommendation by independent double-reading with arbitration based on a real-world dataset of consecutive examinations from a population-based screening program. Using the cutoff with lowest sensitivity loss, the AI system notably increased the proportion of women without false-positive ratings from 11.1 to 38.0% and improved the positive predictive value of recall (PPV-1) from 12.8 to 16.5% as compared to original human reading with arbitration. The improvement was observed at the expense of a sensitivity reduction on the lesion level from 96.7 to 91.1% (17 out of 295 reader-detected malignant lesions were missed by AI).

Several studies demonstrated the ability of AI to achieve better or comparable diagnostic performance to that of an average radiologist [10, 18], to improve radiologists’ performance when used in decision aid in a single-reading setting [3, 5, 6], or to reduce reading workload [4]. As false-positive results are an inherent risk of screening, causing additional diagnostic work-up, anxiety, and distress for the women involved [11, 12], the specificity and PPV-1 are important performance indicators of a screening program [13]. AI might help to increase these parameters.

Consistent with the literature [14], the majority of recalled women in our study sample were relatively young (50–54 years) and had heterogeneous breast tissue (ACR 3). The PPV-1 was 12.8%, which is in the middle field of ranges reported for other European countries (9.8–29%) [15, 22], and in line with the PPV-1 of the national screening program [14].

We focused on a lesion-based AI evaluation. More than 90% of recalled lesions were categorized by readers as low suspicious of malignancy (BI-RADS analogue 4a). The most frequent lesion characteristics (by decreasing frequency) were masses, calcifications, and densities. The majority of malignant lesions presented as calcifications and masses. Using a cutoff of 1, AI missed none of the 44 recalled malignant lesions categorized as highly suspicious (BI-RADS analogue 5). Most of the missed lesions were categorized as BI-RADS 4a (15 of 17) and presented as calcifications (12 of 17).

When focusing on the subgroup of women with mass-related lesions, sensitivity dropped from 97.8 to 96.4% (2 out of 135 reader-detected malignant lesions were missed), while the proportion of women without false-positive ratings increased from 14.2 to 36.7%.

To our knowledge, this is the first study evaluating targeted use of AI as a decision tool to discriminate benign from malignant mammographic lesions to guide indications for further mammography-related assessment. A particular strength of the study is the use of high-quality data of consecutive recalls from a population-based screening program with accurate lesion-based ground-truth labels obtained from standardized assessment and 2-year follow-up for interval cancers.

In a related study of Aboutalib et al. [23], a convolutional neural network was able to discriminate recalled benign mammographic images from negative exams and those with malignancy, suggesting that certain imaging features, potentially not identifiable by human readers, might induce false-positive recalls [23]. Due to the high complexity of deep neural networks, they could currently not further visualize these imaging features, providing a thorough clinical interpretation of them—interpretability of deep learning models is still one of the key challenges in AI research [24]. Furthermore, they did not assess how the performance of their system compares to that of radiologists on the same datasets.

In our study, the lesion localization function of the AI system automatically marked 75.6% of all recalled malignant lesions. Readers should be aware that subtle mammographic lesions found by trained readers are not automatically displayed by AI in about one-fourth of all recalled malignant lesions. Therefore, a thorough reading of screening mammograms by humans is not only required by law, but also necessary in view of the study results. If a suspicious lesion is detected by human reading, the AI system might be used to modify the final decision depending on the lesion-based score. This resulted in 5.8% of the reader-detected malignant lesions to be missed (score 0), but in turn prevented 30.1% of false-positive recalls in our study.

This study is not without limitations. Although we used consecutive screens reflecting the screening population of recalled women in practice, our findings might be limited by the use of retrospective exams of one screening unit. Furthermore, in clinical practice, it can be expected that the AI system would be used to guide the final decision of radiologists to recall a patient, rather than as a stand-alone step on which the decision would be based on. Prospective studies to address this issue are desirable to estimate the effect of using AI in a key decision process of a mammography screening program.

The retrospective study was not designed to test a potential increase in cancer detection or false-positive recalls, as AI was not applied to all cases discussed during consensus conference. The reported performance measures are therefore related to a specific study setting.

In contrast to the AI system used in this study, radiologists had access to prior exams (not processed by the current AI version), if available, that could have impacted their decision to recall a patient. Future software developments as the inclusion of priors might improve the diagnostic performance of the AI system.

In conclusion, the AI system was able to increase discrimination of recalled benign from recalled malignant lesions, thereby increasing the positive predictive value of recall at the expense of some sensitivity loss. Whether this loss of sensitivity can be traded off for a reduction in false-positive recall is an important clinical consideration which cannot be answered by the study as those early-stage cancers might be relevant to improve survival. Application of the AI system to aid the decision to recall a woman seems, however, beneficial especially for masses, where the system reached comparable sensitivity to that of the readers, but with considerably reduced false-positives. Prospective clinical studies are needed to investigate to which extent the potential benefits of AI in breast cancer screening translate into clinical practice.

## Abbreviations

AI: Artificial intelligence
CI: Confidence interval
CR: Computed radiography
DCIS: Ductal carcinoma in situ
PPV-1: Positive predictive value of recall
RSI: Reference standard information

## Declarations

### Guarantor

The scientific guarantor of this publication is Walter Heindel.

### Statistics and biometry

One of the authors (LK) has significant statistical expertise.

### Methodology

• retrospective

• diagnostic study/experimental

• performed at one institution
